# Atlanta Medical and Surgical Journal

**Published:** 1873-04

**Authors:** 


### EDITORIAL AND MISCELLANEOUS.

---

### ATLANTA MEDICAL AND SURGICAL JOURNAL.

### NEW VOLUME—ELEVENTH YEAR.

In presenting the first number of a new volume of the Atlanta Medical and Surgical Journal, we feel it our duty to assure the profession of our earnest desire and determination to make our journal a faithful and comprehensive exponent of medical progress in all the branches of medical science, and a respectable and convenient medium for the physicians of Georgia, and other States, to incorporate the results of their experience and investigations into the medical literature of this country, and become colaborers in the advancement of medicine.

The new additions to our editorial corps, and the systematic division of labor agreed upon under this arrangement, will enable us to bring the most important investigations and discoveries of the leading medical minds of the present age before our readers, and to give them the opportunity and benefit of an early acquaintance with all essential changes in the principles of physiology, pathology and therapeutics, and their influence on practical medicine and surgery.

A firm conviction that the therapeutical principles which govern the treatment of diseases, in order to admit of intelligent and successful practical application, must be the rational consequences snd results of undeniable physiological and pathological conceptions, and that the correctness and success of the practice individually and collectively increases with the clearness of our physiological and pathological comprehensions—or, in other words, that practice is always dependent upon theory as much so as action upon the thought which precedes it—will, we confidently expect, clear us of the reproach of embracing too much of what some may be disposed to term "theory" into the boundaries of our journal, to the exclusion of so-callled practical information. Knowing well enough the importance of empirical observations, we shall accord to them all due importance, and give them cheerfully a place within our paper, as more or less valuable building material in the reconstruction and improvement of the great edifice of medical truth; but as medical science does not suffer so much from a want of observations and facts in pathology and therapeutics, as from an ability of arranging them and classifying them under general points of consideration, for intelligent interpretation and use, for the practical purposes of the profession, we shall always hold that one exposition facilitating the correct comprehension of a train of morbid phenomena is of vastly more practical value to the true physician than the knowledge "by heart" of one dozen recipes said to have been successfully used in that many different diseases.

An earnest endeavor to add to the advancement of rational medicine, in this sense, will certainly not detract, but, we confidently trust, essentially increase the value of the ATLANTA MEDICAL AND SURGICAL JOURNAL with its readers and the profession generally, and we only apprehend that our ability to accomplish good, in this direction, will not prove commensurate with our fervent desire to do so.

